# Findings From the Virginia Cognitive Aging Project: Individual Differences, Well-Being, and Cognition

**DOI:** 10.1093/geroni/igaa057.1984

**Published:** 2020-12-16

**Authors:** Karen Siedlecki

## Abstract

The Virginia Cognitive Aging Project (VCAP) is a cross-sectional and longitudinal study of cognitive functioning in a large sample of healthy community-dwelling adults between the ages of18-99 years (Salthouse,2009). Data are collected on several domains of cognitive functioning and subjective ratings of cognition, as well as a myriad of individual difference characteristics including self-reports of physical activity, cognitive activity, social support, personality, well-being, and affective measures. This symposium focuses on findings from VCAP that examine cross-sectional and longitudinal links between individual difference characteristics, indicators of well-being, and objective and subjective cognition. These topics include the cross-sectional assessment of >5,000 participants on the mediating role of Need for Cognition on the relationship between cognition and well-being (Yazdani & Siedlecki) and the relationship between social support and ratings of subjective cognition (Mueller & Minahan). Jung uses cross-lagged analyses to assess temporal relationships between physical and cognitive activity and cognition. Falzarano et al. present findings regarding the longitudinal relationship between subjective and objective measures of cognition. Finally, Minahan and Siedlecki present findings examining the temporal relationship between ratings of loneliness and depression over time. The symposium provides insights into the complex role of individual differences characteristics and cognitive functioning across the adult lifespan.

